# Supplementary material for: Gating mechanism of the extracellular entry to the lipid pathway in a TMEM16 scramblase
Source: Nat Commun. 2018 Aug 14;9:3251. doi: 10.1038/s41467-018-05724-1 (PMC6092359; doi:10.1038/s41467-018-05724-1)
Supplement: Supplementary file 1 — Supplementary Information [file 41467_2018_5724_MOESM1_ESM.docx]

**Gating mechanism of the extracellular entry to the lipid pathway in a TMEM16 scramblase**

**Byoung-Cheol Lee, George Khelashvili, Maria Falzone, Anant K. Menon, Harel Weinstein and Alessio Accardi**

**SUPPLEMENTARY INFORMATION**

**Supplementary Note 1**

In the tICA landscape shown in Supplementary Figure 14c, the region denoted by the yellow dot indicates the origin of all Stage 3 trajectories, where a lipid partitions into the groove from the IC leaflet and moves some way towards the EC leaflet (Supplementary Figure 13b). The tICA space contains two partially overlapping regions (Supplementary Figure 14d) containing, respectively, states of the system evolving towards a complete flip (Supplementary Figure 14d, left panel, “Flipped”), and states where no such transition occurs and the lipid either remains within the groove or diffuses back into the IC leaflet (Supplementary Figure 14d, right panel, “Not Flipped”). Thus, the tICA space captures the key mechanistic steps that define the transition of a lipid between the two leaflets (Fig. 7a). To facilitate visualization, we show structural representations corresponding to key intermediate conformations along the transition pathway (Fig. 7a, States 1-6). The histograms in Fig. 7c quantify dynamic variables in the 6 States identified structurally. This information, supplemented by the histograms in Supplementary Figure 15b, characterizes the changes occurring in the system that evolves through the steps of lipid transfer between the leaflets.

Specifically, in State 1 the lipid is in the IC vestibule of the groove, with its headgroup far below R432 (Z=-20 Å in Fig. 7c, “Lipid-R432”) and its tail relatively upright (small tilt values in Fig. 7c, “Lipid Orientation”). In this State, the EC part of the groove is in closed conformation as quantified by the pair-wise distances between the gate residues (see “T333-Y439” in Fig. 7c, “E313-R432” and “E318-R432” in Supplementary Figure 15b) as well as by the overall distance between the EC ends of TM3 and TM6 helices (Fig. 7c, “TM3-TM6 EC”).

States 2-3 comprise the initial stages of the lipid flip, as its headgroup moves about half-way towards the EC leaflet (Fig. 7c, “Lipid-R432”) while the tail assumes a horizontal orientation (Fig. 7c, tilt values of ~90°). During these stages, the EC constrictions remain closed (see the histograms of the EC gate variables in Fig. 7c and in Supplementary Figure 15b). The next stage of the lipid translocation (State 4) is characterized by the opening of the T333-Y439 constriction (Fig. 7c), which allows the lipid headgroup to progress further towards the external leaflet (“Lipid-R432” distance in Fig. 7c reaches Z=0Å) as its tails begin to re-orient (tilt angles of ~140° in Fig. 7c). In addition, the E313-R432 and E318-R432 interactions begin to destabilize (Supplementary Figure 15b) resulting in the emergence of a population of states with a relatively wide EC vestibule (see “TM3-TM6 EC” in Fig. 7c).

As the lipid moves past the T333-Y439 constriction it completes the flip and the headgroup reaches the level of the R432 residue (State 5 in Fig. 7c). Concomitantly, the TM3-TM6 gates are more frequently in open configurations (Fig. 7c, Supplemental Fig. 15b). Note that in this state the lipid head-group is still coordinated by the groove residues (see “Lipid Coordination” in Supplementary Figure 15b). This coordination is lost in the last stage of the flipping process (State 6, “Lipid Coordination” in Supplementary Figure 15b) when TM3 and TM6 disengage completely from each other (Fig. 7c) allowing the lipid to leave the groove and diffuse into the external leaflet (see “Lipid-R432” in Fig. 7c).


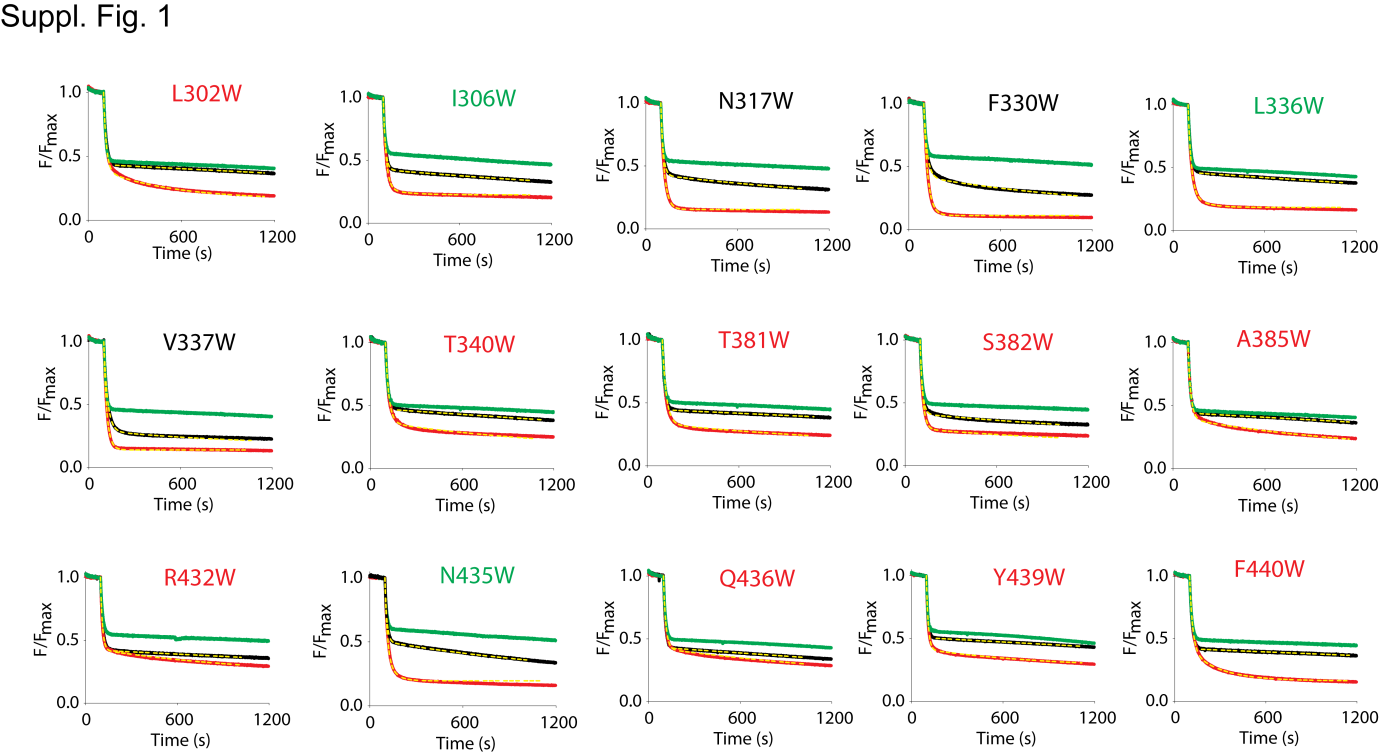


**Supplementary Figure 1. Effect of tryptophan mutants on scrambling.** Time course of dithionite-induced fluorescence decay for the indicated nhTMEM16 mutants in the presence (red) and absence (black) of Ca^2+^. Dashed yellow lines indicate fits to Eq. 1. Protein-free traces are shown in green.


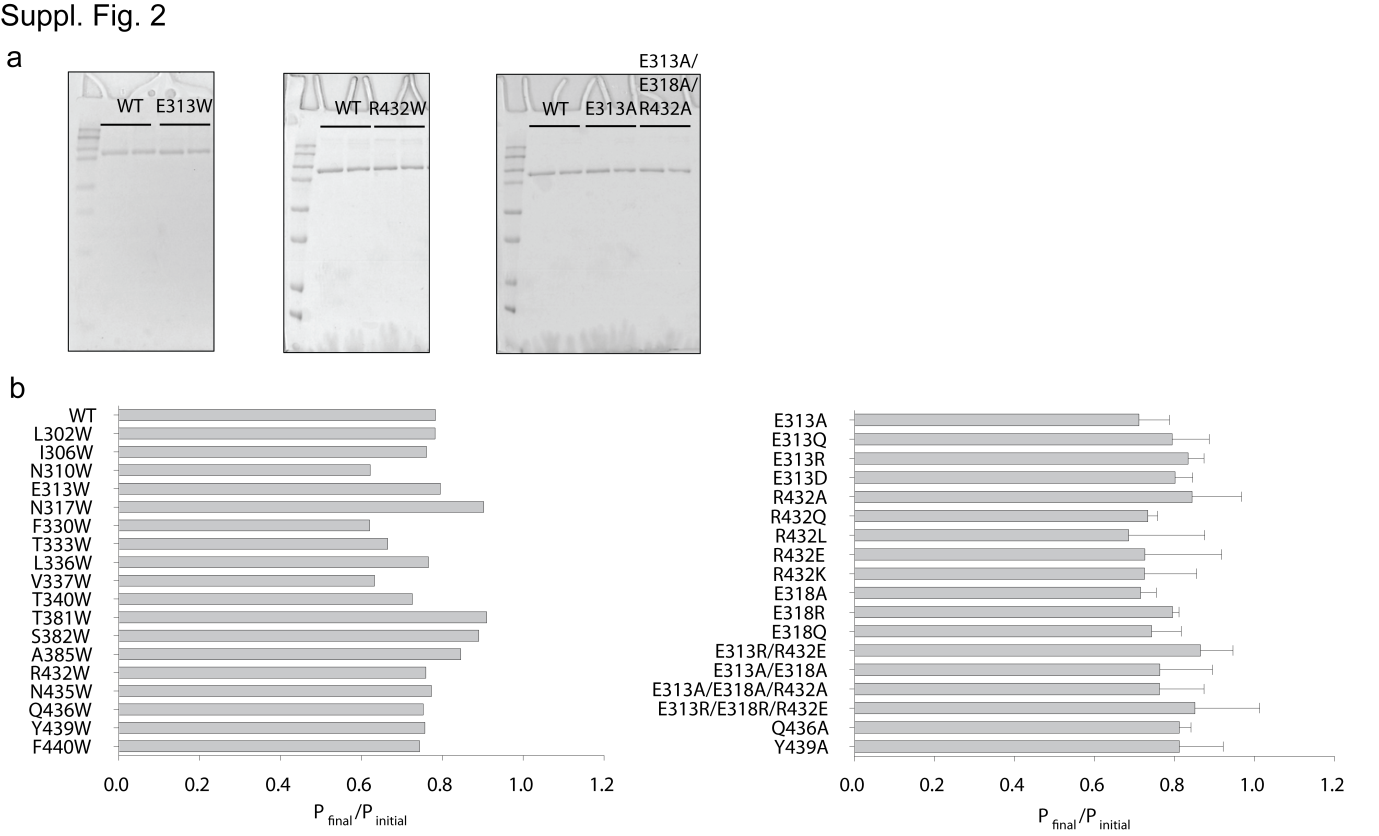


**Supplementary Figure 2. Reconstitution of WT and mutant nhTMEM16 into proteoliposomes.** a) Coomassie stained SDS gels showing protein incorporation into liposomes for WT (all 3 panels) and E313W (left), R432W (middle) and E313A and E313A/E318A/R432A (right) nhTMEM16. b) Quantification of the fraction of WT and mutant nhTMEM16 protein reconstituted in proteoliposomes for all mutants. Left panel, n=1; right panel n>2.


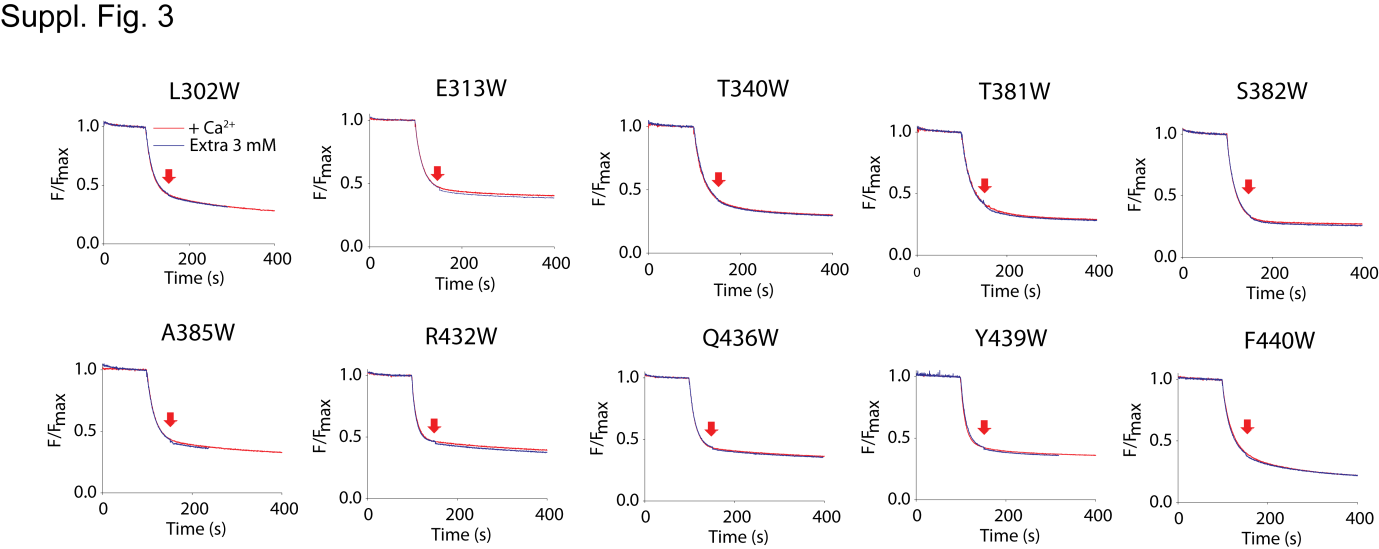


**Supplementary Figure 3. The mutants do not reduce the apparent Ca^2+^ affinity.** Time course of dithionite-induced fluorescence decay for the indicated nhTMEM16 mutants in the presence of 0.5 mM Ca^2+^ (red). Blue traces indicate experiments where 3 mM Ca^2+^ was added to the cuvette 100 s post-dithionite addition (red arrow).


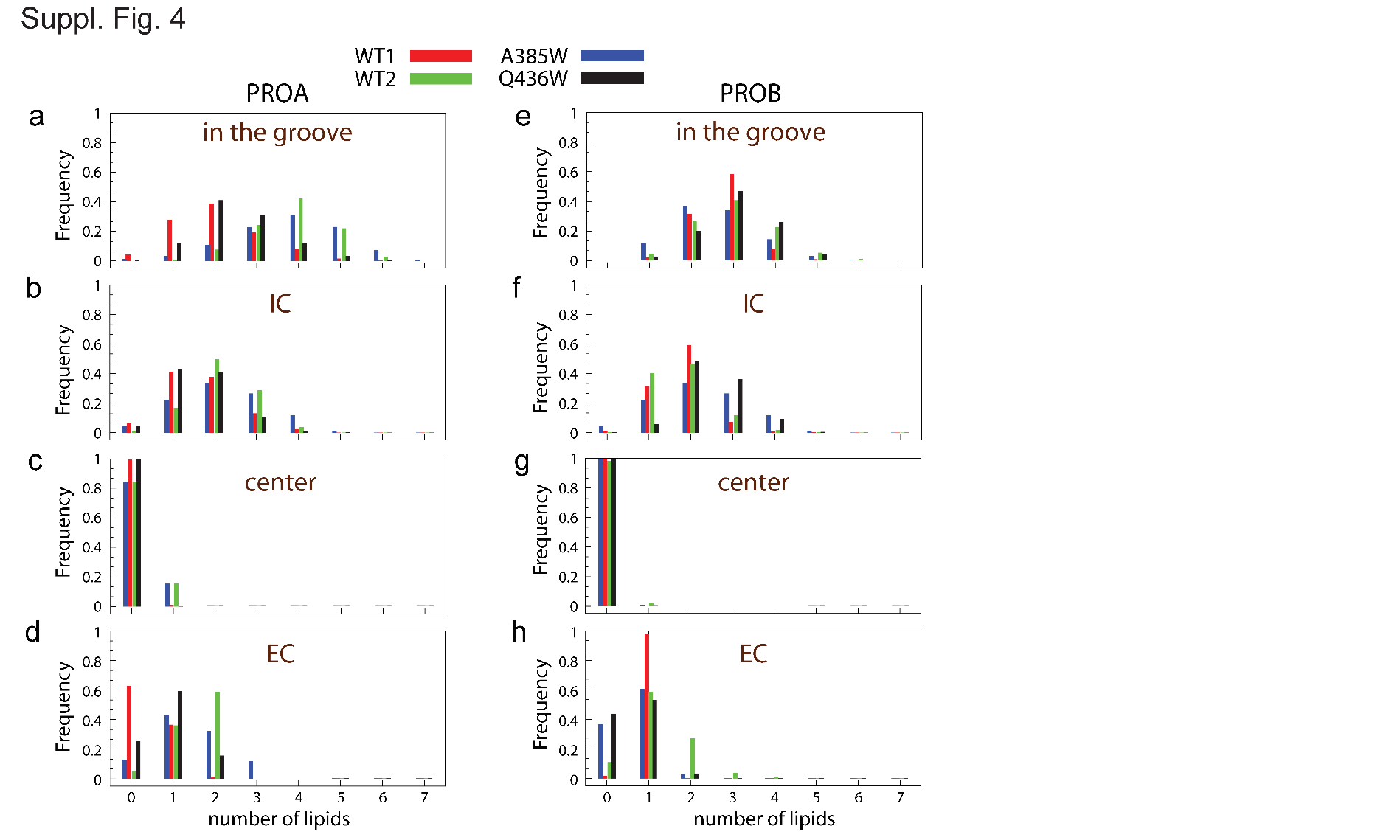


**Supplementary Figure 4.**  **Simultaneous occupancy of the hydrophilic groove of nhTMEM16 by lipid headgroups.** The frequency of finding a specific number of lipid headgroups simultaneously occupying the groove and its different compartments (IC side of the groove, center of the groove, and EC side of the groove) in the WT1 (red), WT2 (green), A385W (blue), and Q436W (black) simulations (see Methods for the definition of the groove and its different regions). The data are shown separately for each protomer of nhTMEM16 (denoted as PROA, panels a-d, and PROB, panels e-h).


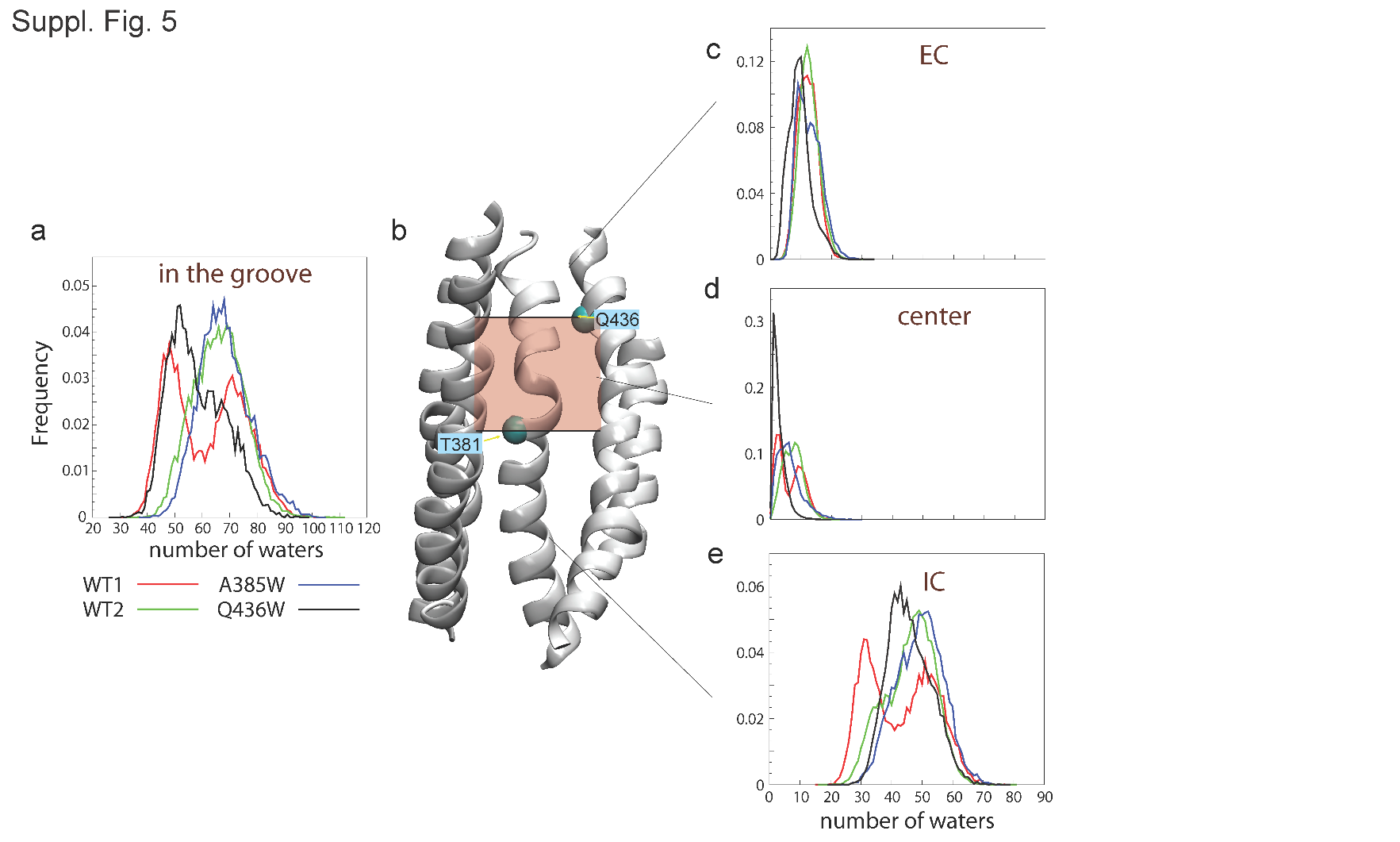


**Supplementary Figure 5.**  **Water occupancy of the hydrophilic groove of nhTMEM16.** The frequency of finding a specific number of water molecules in the groove (a), and its three compartments (EC side of the groove, center of the groove, and IC side of the groove, panels c-e) calculated from the WT1 (red), WT2 (green), A385W (blue), and Q436W (black) simulations (see Methods for the definition of the groove and its different regions). For each construct, the data shown are the average over the two protomers of the nhTMEM16. Panel (b) shows a snapshot of the nhTMEM16 (TM helices 3-7) from WT2 simulation highlighting the location of the central region of the groove devoid of lipids (the shaded rectangle). The thick black lines surrounding the shaded rectangle indicate the z-axis positions of the Cα atoms of residues T381 and Q436 (cyan spheres). See also Figure 4b in the main text.


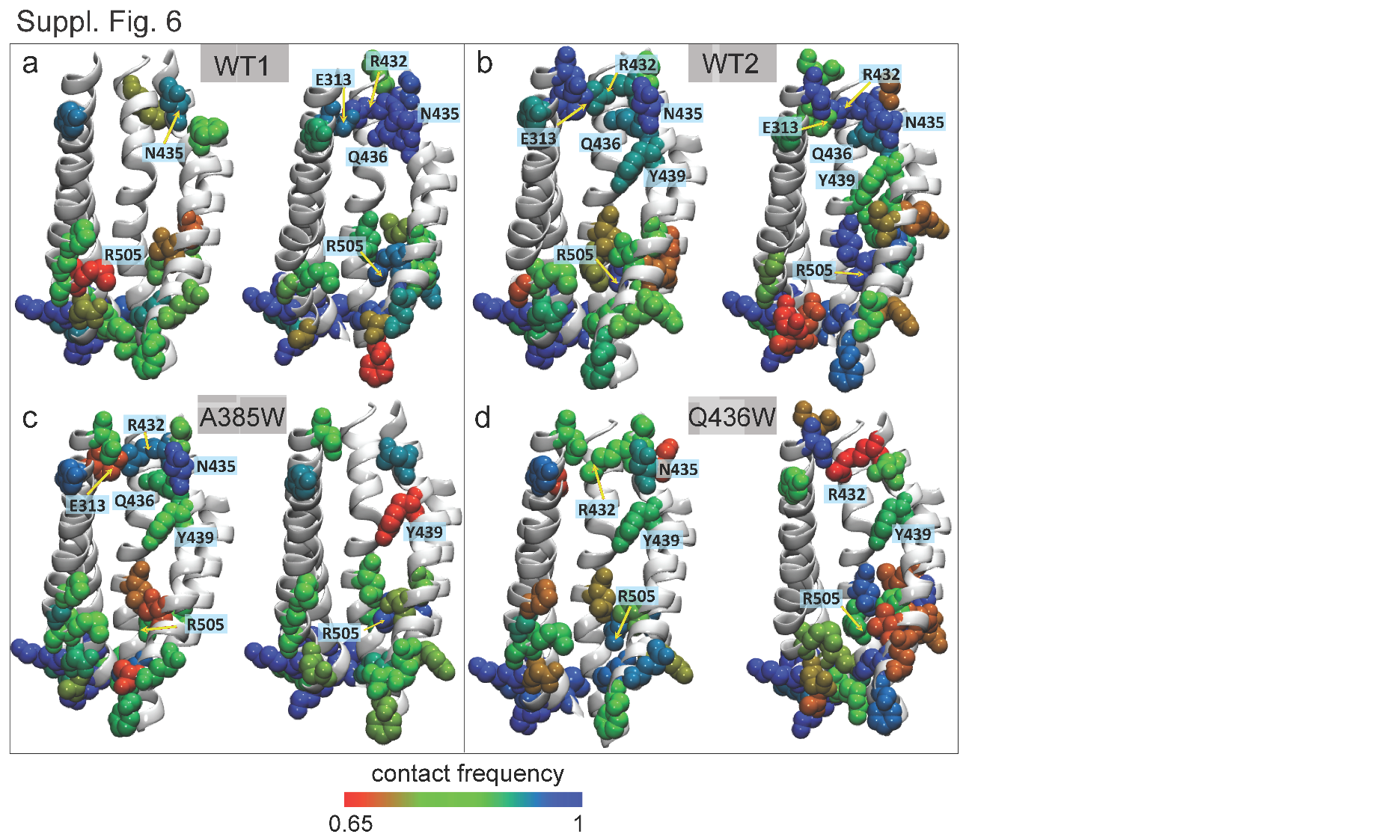


**Supplementary Figure 6. Lipid contact heat maps.** The TMs 3-7 section of the nhTMEM16 structure is shown, with highlighting of groove residues (in van der Waals and colored) that are found to be in contact with a lipid headgroup 65% of the time in the trajectories for WT1 (a), WT2 (b), A385W (c), and Q436W (d) (shown separately and for each protomer). A lipid headgroup was considered in contact with a residue if the phosphate atom of the lipid was within 7Å of any atom of the residue.


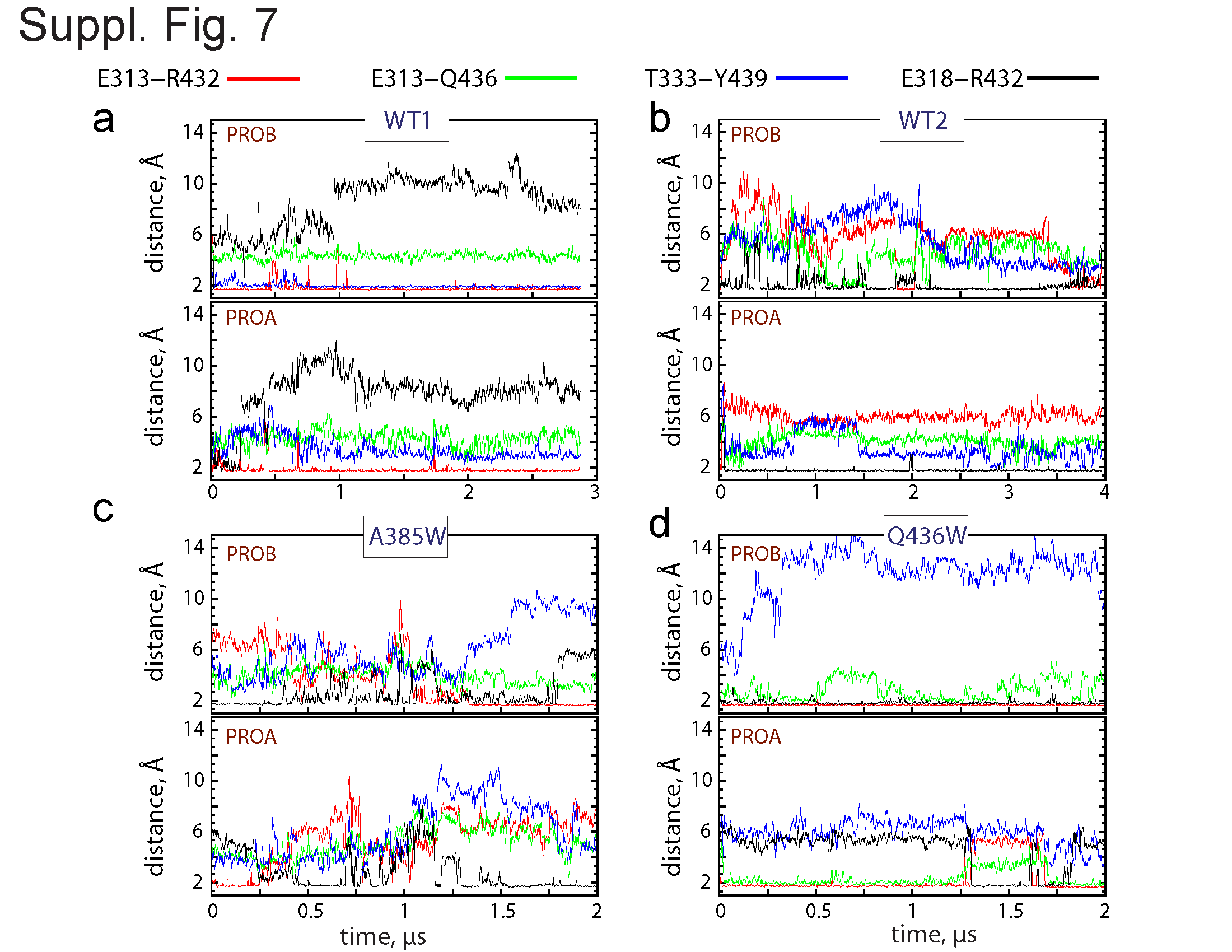


**Supplementary Figure 7. Dynamic rearrangement of the network of polar residues at the extracellular side of the groove.** Time evolution of distances in the pairs E313-R432 (red), E313-Q436 (green), T333-Y439 (blue), and E318-R432 (black) in the MD trajectories of the various nhTMEM16 constructs (panels a-d show data from WT1, WT2, A385W, and Q436W trajectories, respectively). For a particular system, data for the two protomers are presented in separate panels (top and bottom). For the Q436W system, the green traces represent the E313-W436 distance.

**Supplementary Figure 8. Coordination of lipids by the R432/E313 pair of residues.** Histograms show the number of lipids simultaneously coordinated by the R432/E313 pair of residues in the WT1 (red), WT2 (green), A385W (blue), and Q436W (black) simulations. PROA and PROB are the protomers of each construct. A lipid was considered coordinated by the R432/E313 residue pair if its phosphate atom was within 5Å of any atom of these residues.


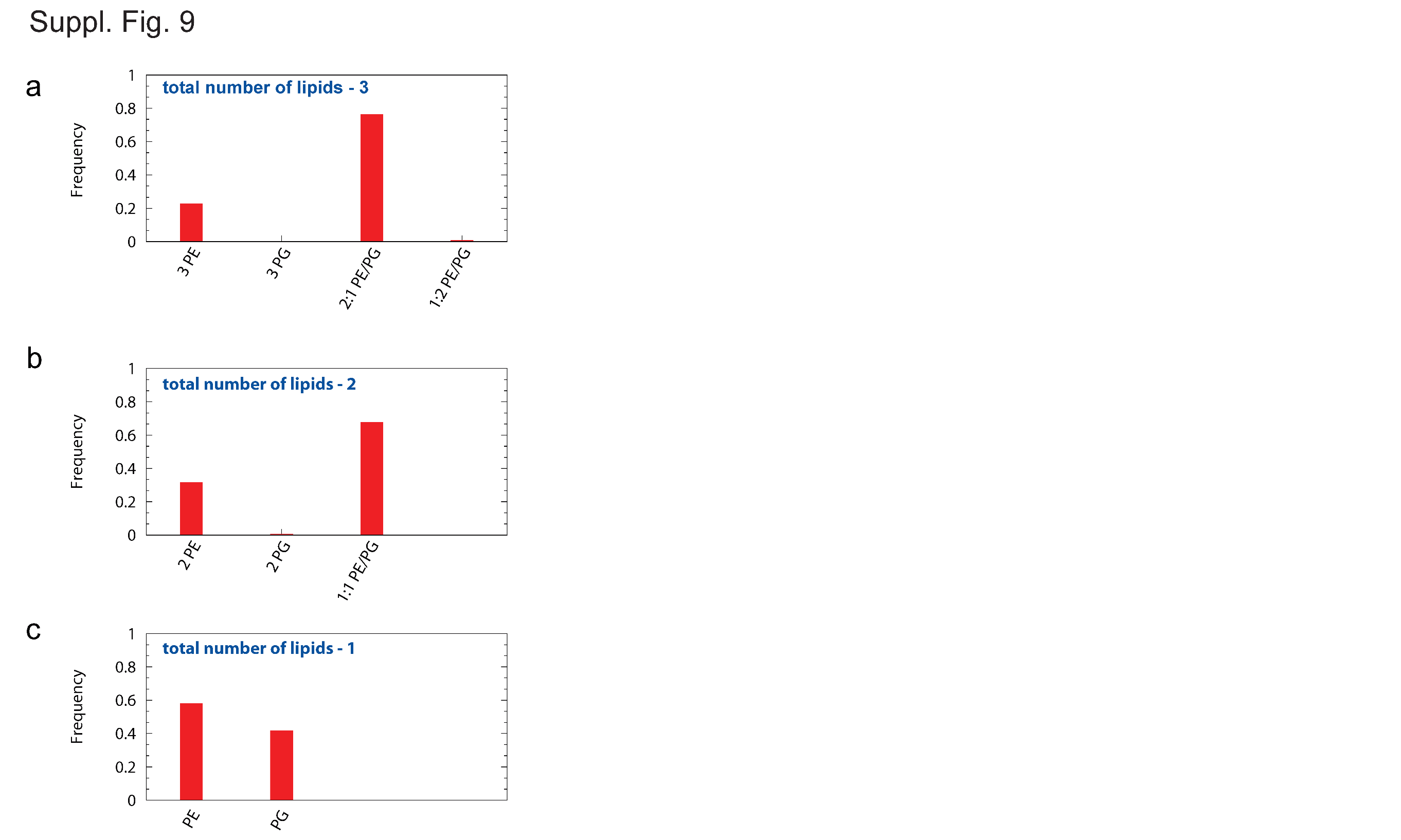


**Supplementary Figure 9. Specificity of lipids accumulating near the E313/R432 pair of residues.** The panels present the frequencies of finding PE and PG headgroups near the E313/R432 pair when the total number of lipids at the extracellular side of the groove is either 3 (a), or 2 (b), or 1 (c). A lipid is counted if its P atom is within 5Å of any atom of the residue pair. For each panel, the occurrence of all the possible combinations of PE:PG ratios are counted and the data is normalized (separately for each panel) to the total number of events with 1, 2, or 3 lipids, respectively. The data were generated by combining all the trajectories.


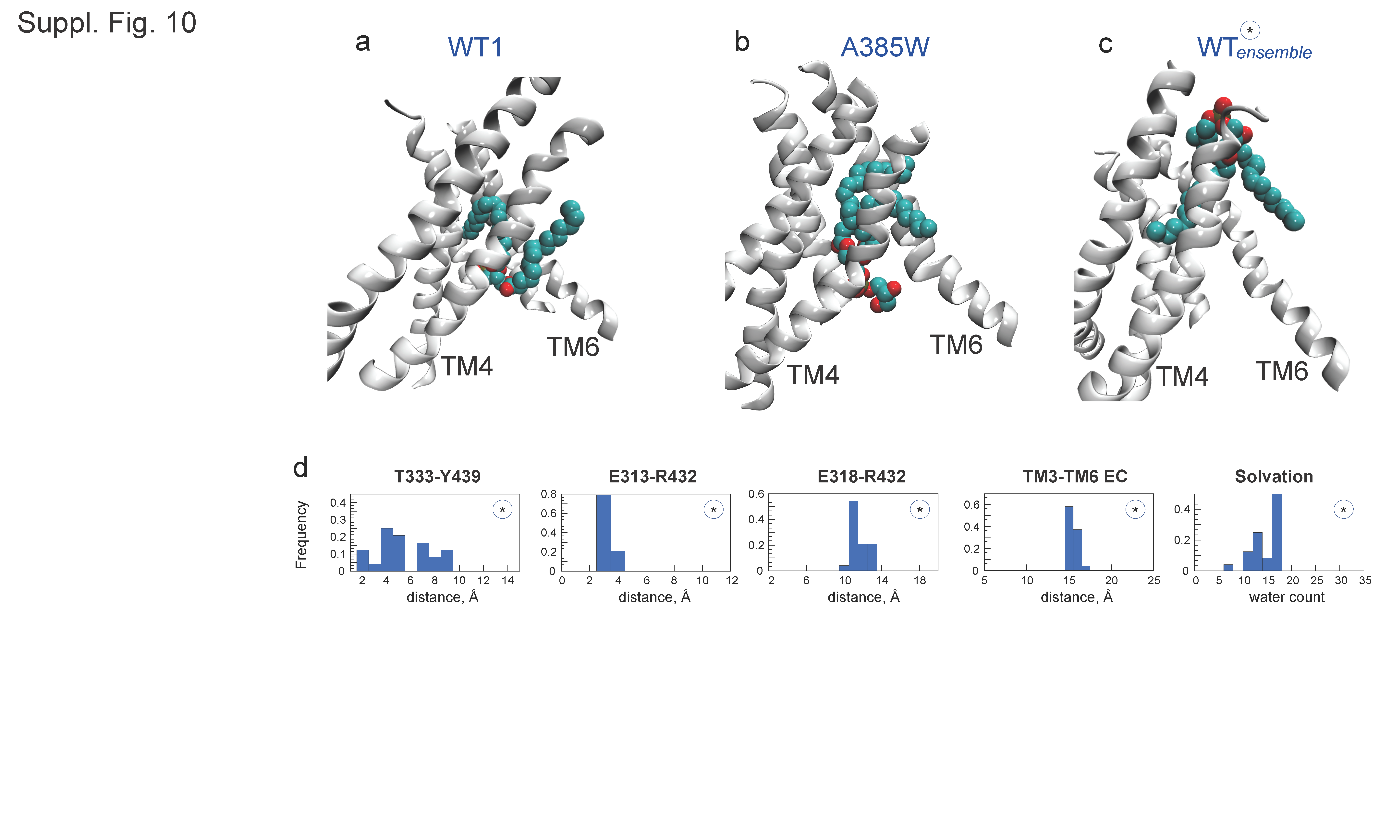


**Supplementary Figure 10. Rare mode of lipid penetration into the groove. (a**-**c)** In this mode, the penetrating lipid (rendered in van der Waals) inserts both its headgroup and tail into the groove of the nhTMEM16 in the WT1 (**a**), A385W (**b**), and WT*^ensemble^* (**c**) simulations. TM4 and TM6 are labeled. (**d**) Structural characteristics of the microstate describing the rare mode of lipid penetration in the WT*^ensemble^* simulations (see Fig. 7a, Microstate denoted by “*”): the first 3 panels from left to right record the probability distributions of the T333-Y439, E313-R432, and E318-R432 distances; the next two panels show probability distributions of the opening between the EC ends of TM3 and TM6 (measured as the distance between the centers-of-mass of two groups: C_α_ atoms of residues 315 to 318 on TM3, and C_α_ atoms of residues 432 to 435 on TM6), and of number of water molecules in the EC vestibule (see Methods for definitions of the different parts of the groove).


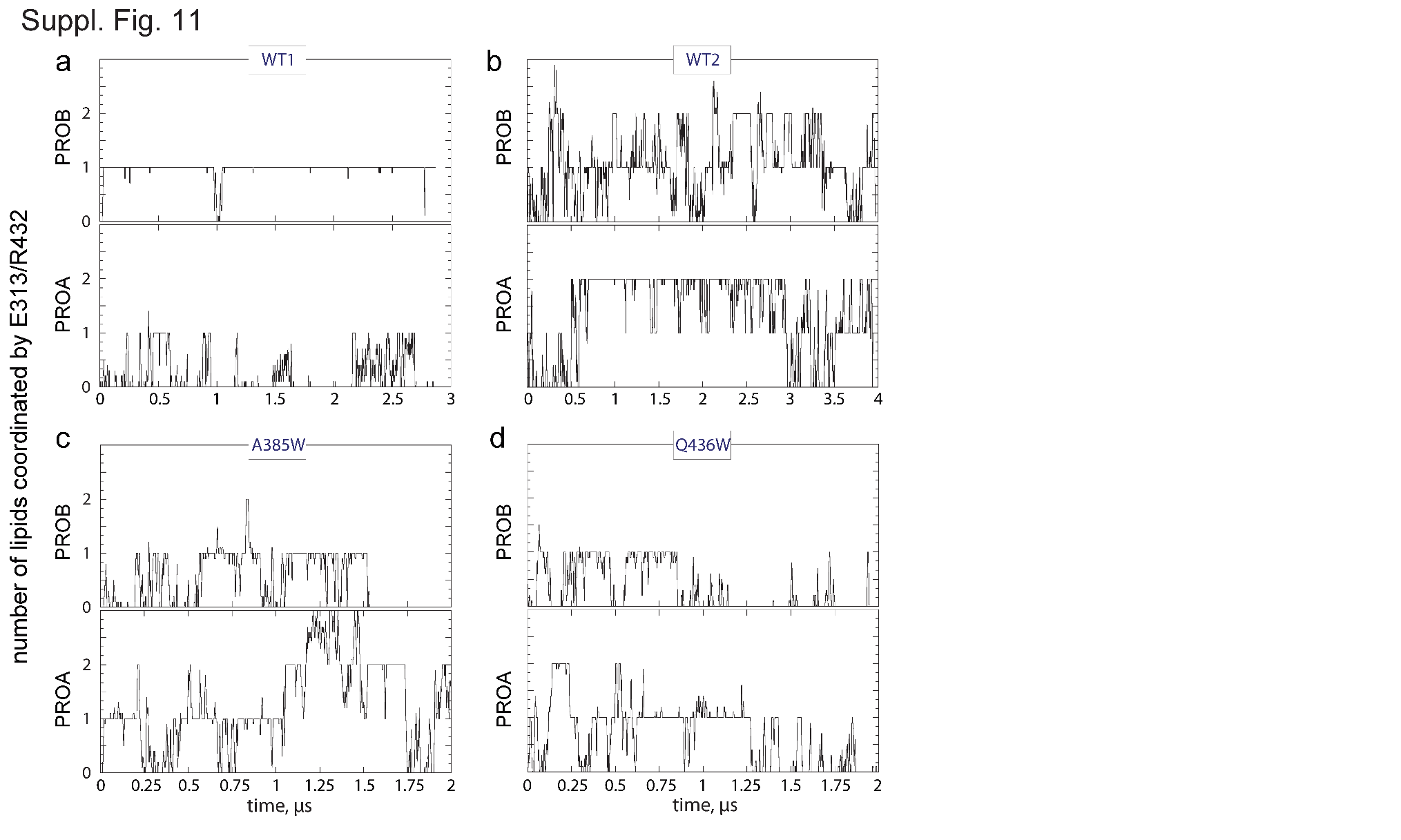


**Supplementary Figure 11. Accumulation of lipids near the E313/R432 pair.** Time traces of numbers of lipid headgroups within 5Å of the E313/R432 pair of residues from the simulations of the various nhTMEM16 constructs (data from WT1, WT2, A385W, and Q436W trajectories are shown in panels A through D, respectively). For a particular system, data for the two protomers are presented in separate panels (top and bottom).


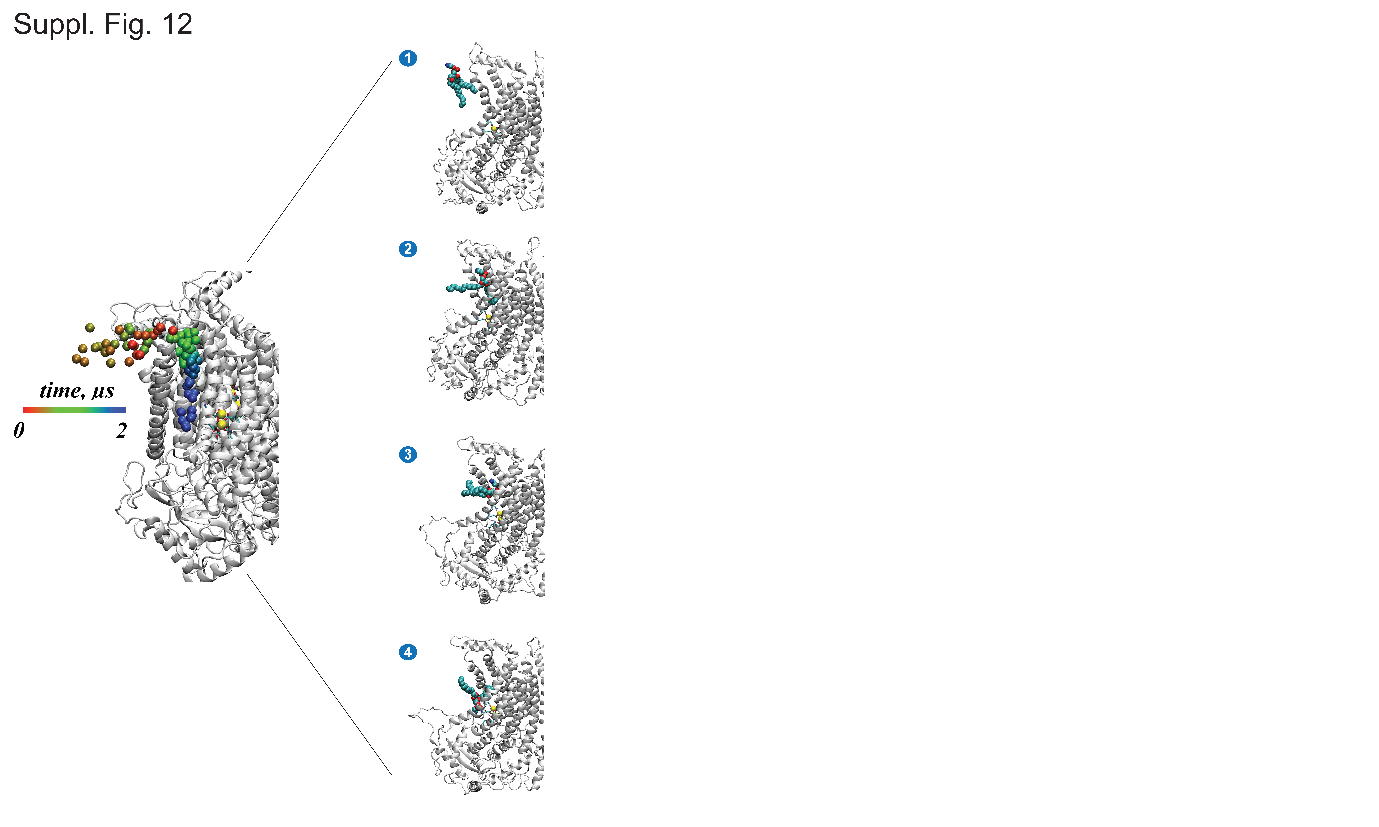


**Supplementary Figure 12. Lipid flipping event at the end of transfer observed in the MD simulations of A385W nhTMEM16.** (*left*)Time evolution of the position of the phosphate atom of the scrambled lipid in the simulation. The color code of the spheres is shown in terms of simulation time: blue spheres indicate positions at the end of the trajectory. The lipid partitions in the EC side of the groove at ~1 µs into the trajectory (green color). (*right*) Snapshots of the system illustrating positioning and arrangement of the transferred lipid inside the hydrophilic groove at various time points along trajectory: (1) The lipid is on the extracellular side and in the bulk; (2) Initial partitioning of the lipid head group into the groove; (3) The lipid starts translocation through the groove; (4) The lipid is flipped with its headgroup merging with the intracellular leaflet.


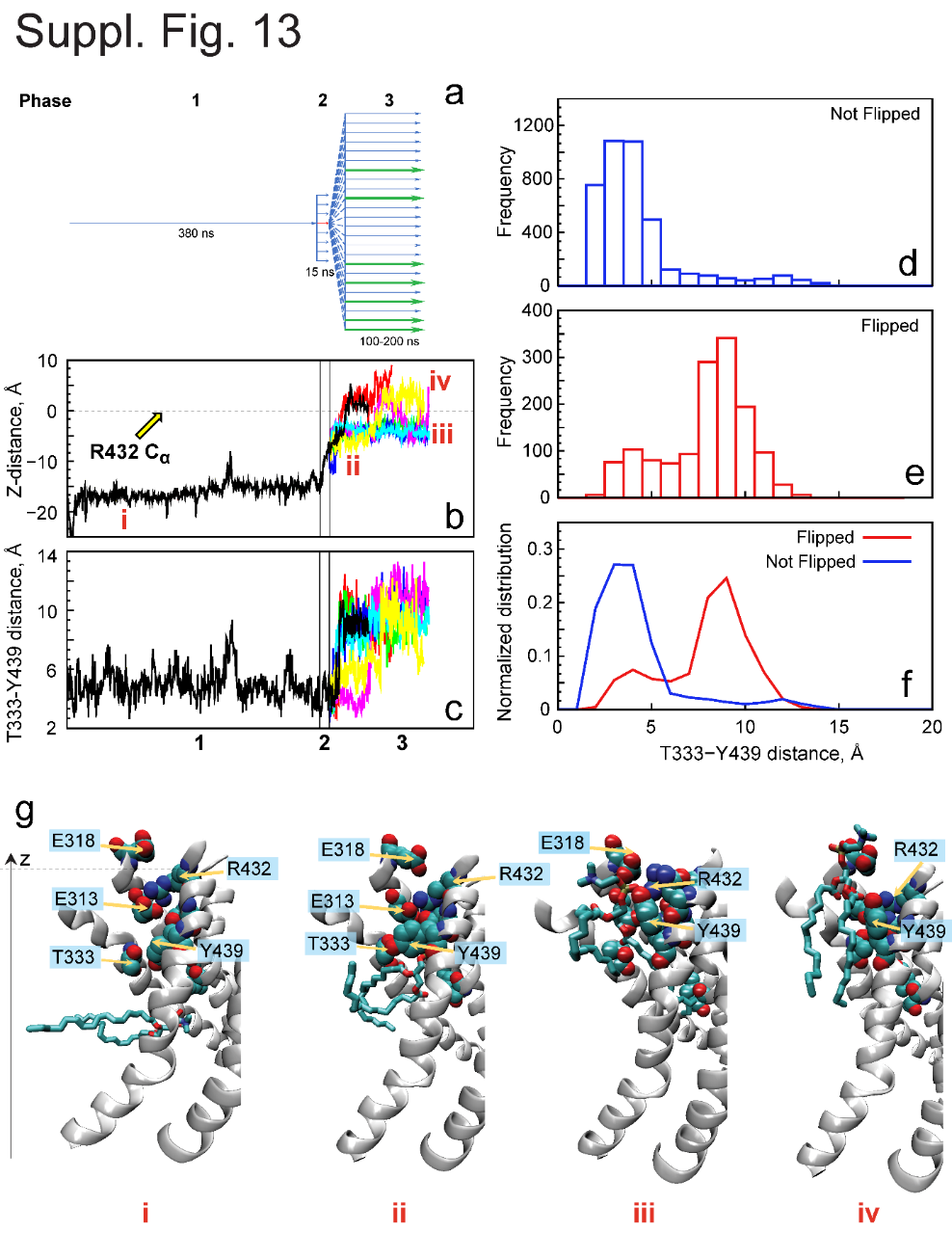


**Supplementary Figure 13. (a)** Multi-stage iterative MD simulation protocol used for unbiased atomistic MD simulations of the wild type nhTMEM16 in POPC lipid membranes (see Methods, Table 3). The simulations were carried out in three stages. Stage 1 is a single simulation of 380ns time-length. Stage 2 consists of 8 statistically independent 15ns simulations from random velocity resets of the last frame of the Stage 1. Stage 3 is composed of 24 statistically independent simulations (obtained again by randomizing the velocity field), initiated from the Stage 2 simulation (indicated by red arrow), in which lipid translocation occurred from the IC side to the central region of the groove. On 100-200ns time-scales, the advancing lipid was observed to complete the flip to the EC side in 7/24 simulations (denoted by green arrows). (**b**-**f**) Opening of the T333-Y439 constriction is necessary to trigger lipid translocation from the IC to the EC leaflet. Time-evolution in simulation Stages 1-3 of (**b**) the Z-directional distance between the phosphorus atom of the translocated lipid and the Cα atom of R432; and (**c**) the T333-Y439 minimum distance. The three stages are separated by vertical lines. In panel **a**, the distances on the Y-axis are relative to the “0” position of the lipid with its phosphorus atom aligned in the Z direction with the Cα atom of R432. For Stage 2, the data are shown only for the trajectory in which the lipid advances from the IC side to the central region of the groove (see panel “**a**”). For Stage 3, results are shown only for those trajectories (different colors) in which the complete flip of the translocated lipid was observed. (**d-e**) Histogram of the T333-Y439 minimum distance in the Stage 3 simulations plotted separately for the combination of trajectories in which the lipid flipped (**d**), and for the ones in which the flip was not observed (**e**). Panel (**f**) presents the results from the histograms in **c** and **d** normalized by their respective overall number of analyzed frames. (**g**) Structural representation of the system at different time-points along the trajectories, denoted by i-iv in correspondence to the regions indicated in panel **a**. The snapshots representing Stage 3 simulations were generated from 6 replicate trajectories. The advancing lipid is rendered in licorice, and relevant groove residues are shown in space fill representations and labeled.


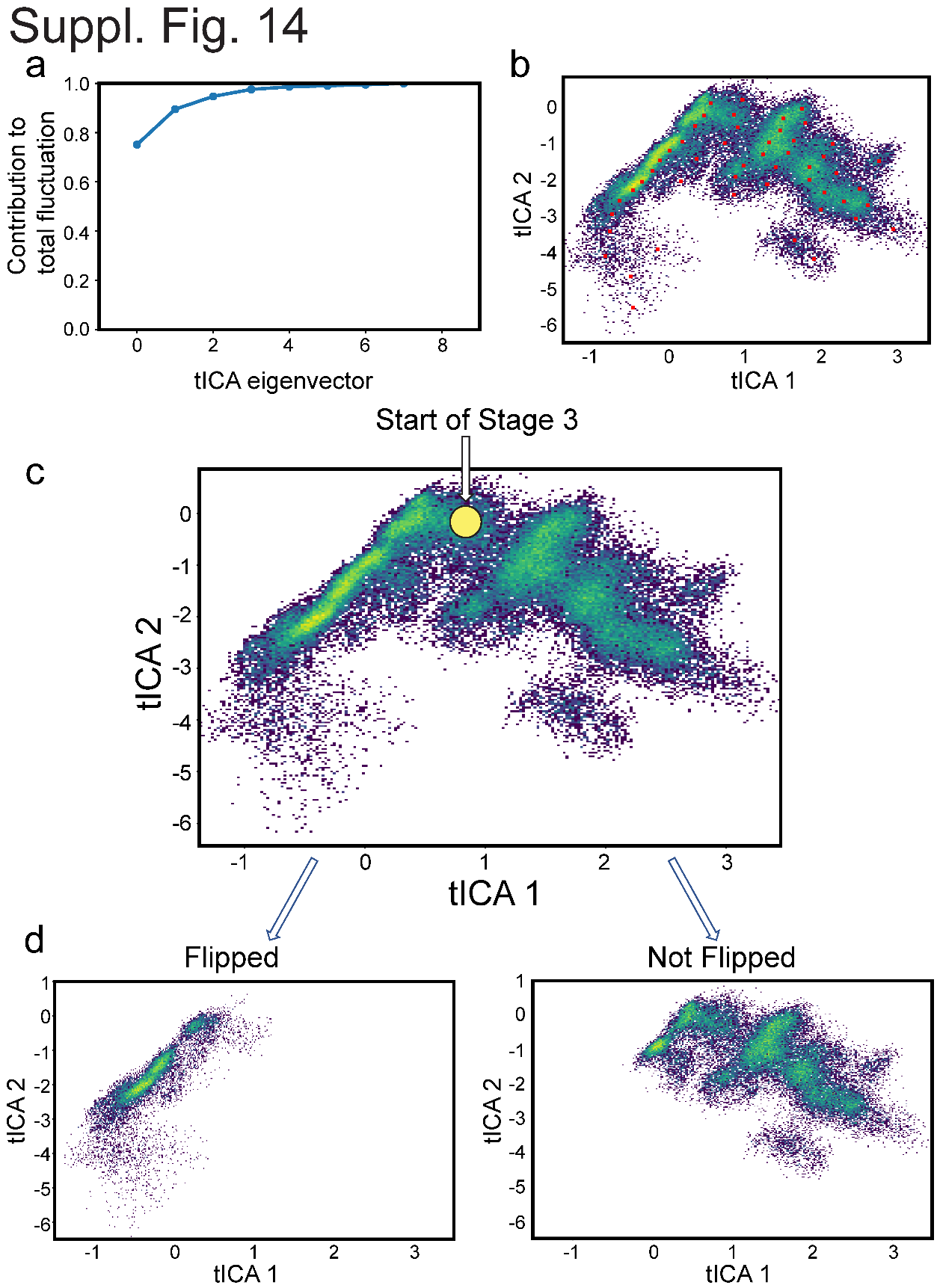


**Supplementary Figure 14. Dimensionality reduction with the tICA approach.** (**a**) Contribution of all eight tICA eigenvectors to the total fluctuation of the system. (**b**) All Stage 3 trajectories mapped on the 2D landscape of the first two tICA eigenvectors (tICA 1 and tICA 2). Shown also are locations of the 50 microstates (red squares) obtained from the clustering analysis of the conformational space (see Methods). (**c**) The 2D tICA landscape from panel **b** highlighting the location of the initial conformation of the system (yellow circle) from which Stage 3 simulations were initiated. (**d**) Projection onto the 2D tICA landscape from panel 3 of all the Stage 3 trajectories in which lipid flip was observed (*left*) and in which lipid did not flip (*right*).


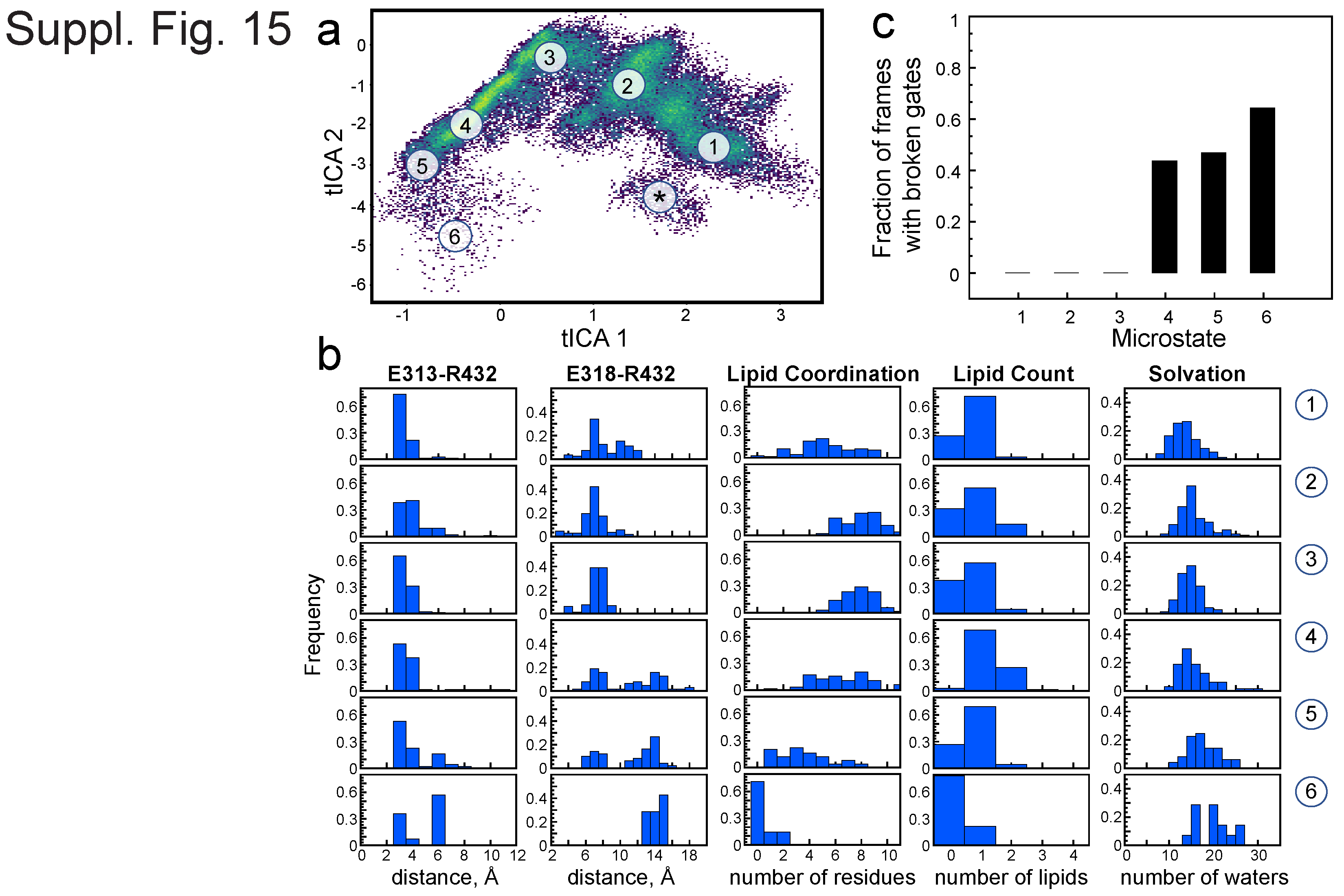


**Supplementary Figure 15. Structural characterization of the tICA landscape provide mechanistic insights into lipid scrambling process.** (**a**) The 2D tICA landscape from Fig. 7a denoting the location of microstates 1-6 that represent the translocation of the lipid through TMEM16 (Microstate denoted by “*” captures rare mode of protein-lipid interaction in which the hydrocarbon tail of a lipid inserts into the EC side of the groove interfering with the translocation of the advancing lipid). (**b**) Structural characteristics of the selected 6 microstates: the first 2 columns from left to right record the probability distributions of the distances E313-R432, and E318-R432 in each microstate; the next column quantifies lipid coordination by the protein (defined as number of protein residues within 4Å of any atom of the head-group of translocating lipid; the head-group includes all the atoms from N to O11 in CHARMM atom-name nomenclature); the last 2 columns show the counts of lipid headgroup and waters, respectively, in the EC vestibule of the groove in each microstate (see Methods for definitions of the different parts of the groove) (**c**) The plot compares the fractions of trajectory frames in which the three gates, T333-Y439, E313-R432, and E318-R432, are simultaneously broken in the microstates (the T333-Y439 gate was assumed to be broken if the T333-Y439 distance was > 8Å (see Fig. S13c-f); the E313-R432 and E318-R432 gates were assumed to be broken if the distance between carbonyl oxygen of Glu and sidechain nitrogen of Arg was > 4Å).


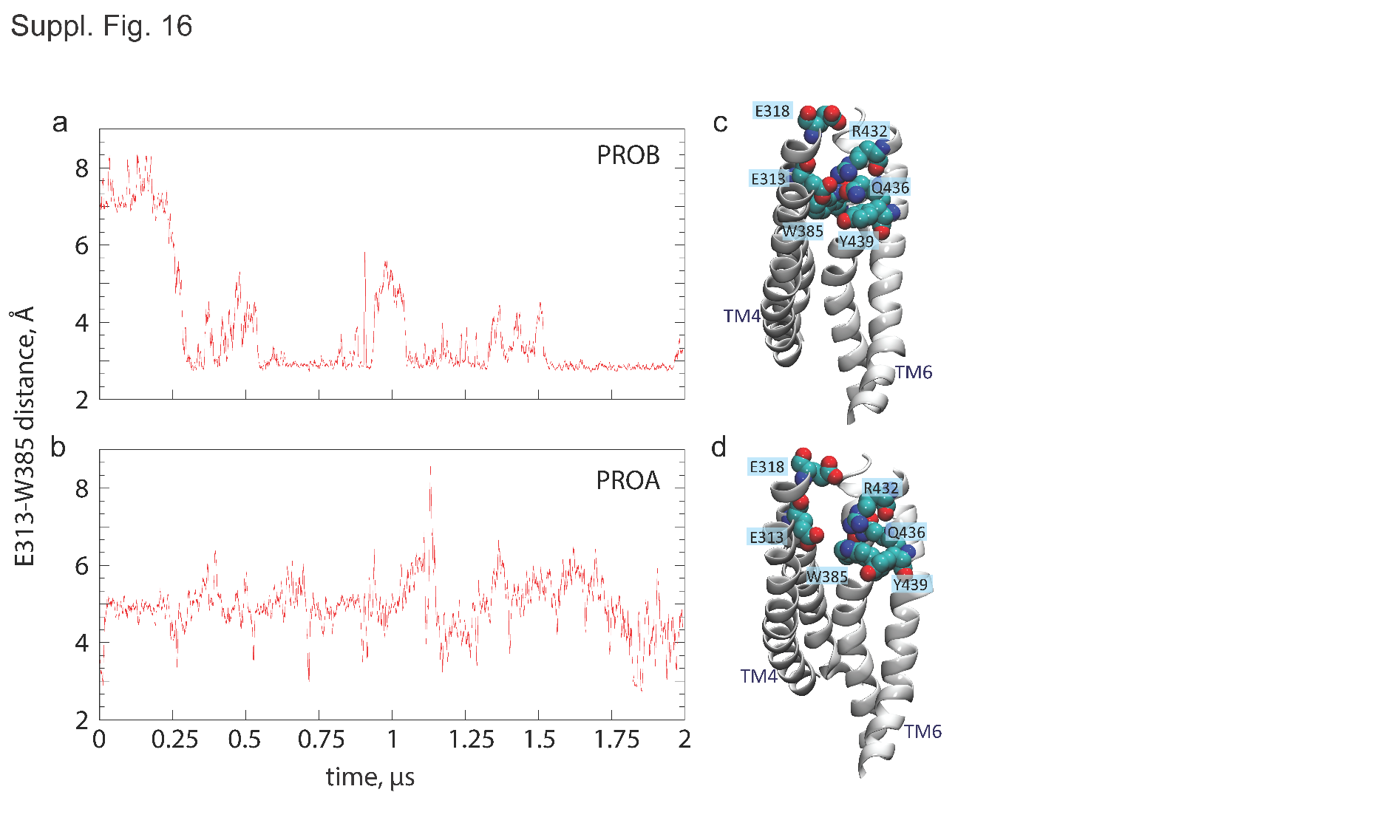


**Supplementary Figure 16. The Trp replacing A385 can hydrogen bond with residue E313. (a**-**b**) Time evolution of the minimum distance between E313 and W385 in the MD simulation of the A385W nhTMEM16 construct. The two panels show data for the two protomers of the protein (PROA and PROB). (**c**-**d**) Snapshots of nhTMEM16 A385W showing conformations of the system when the hydrogen bond between E313 and W385 is present (**c**), or broken (**d**). Locations of the TMs 4 and 6 lining the hydrophilic groove, as well as structural re-arrangement of several key residues in the groove (in van der Waals and labeled) are also illustrated.


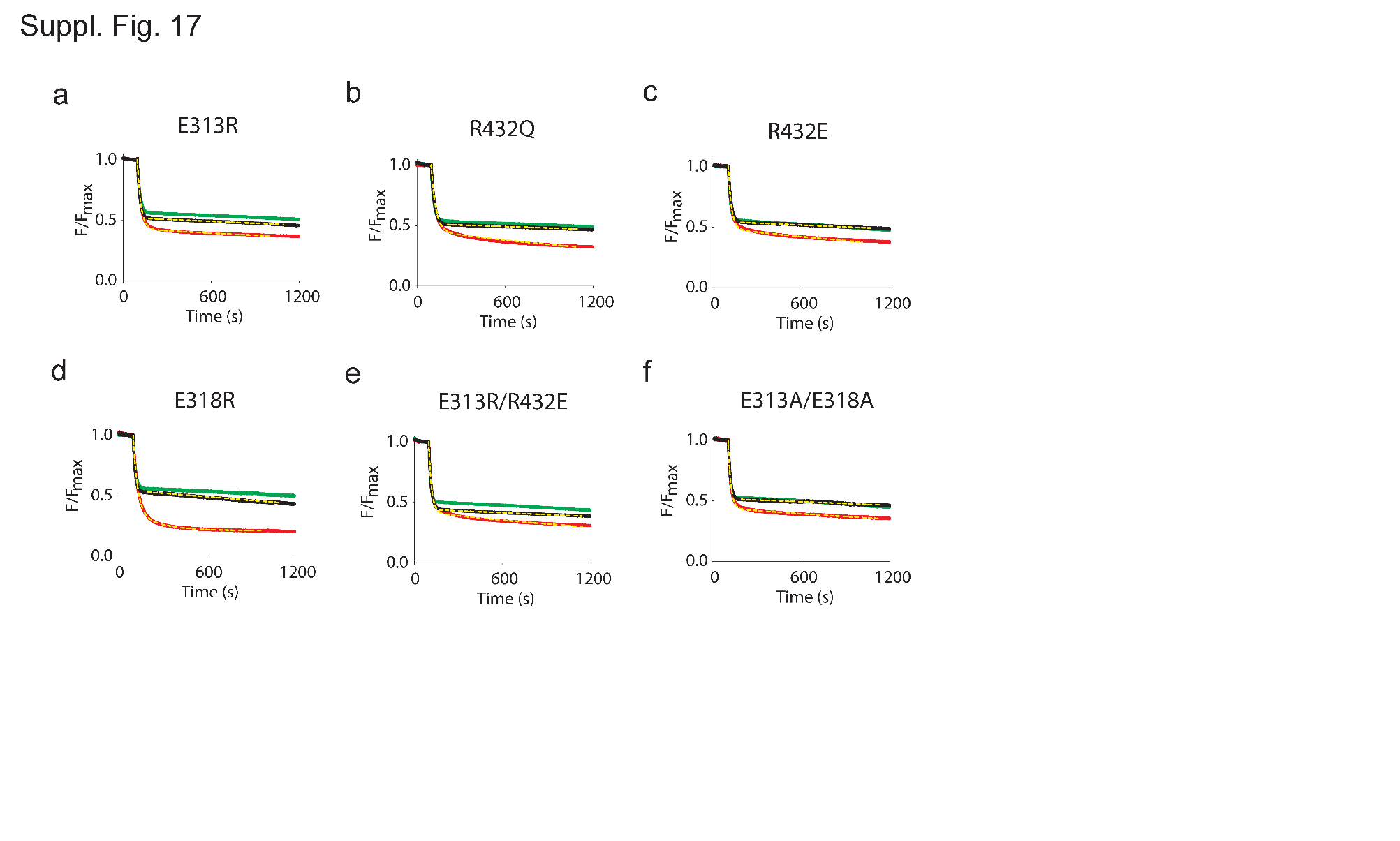


**Supplementary Figure 17. Functional characterization of the gate mutants.** Time course of dithionite-induced fluorescence decay for the indicated nhTMEM16 mutants in the presence (red) and absence (black) of Ca^2+^. Dashed yellow lines indicate fits to Eq. 1. Protein-free traces are shown in green.

**Supplementary tables**

| Protein | [Ca^2+^] (mM) | α (s^-1^) | β (s^-1^) | f_0_ | γ (s^-1^) | N(s) | A (%) | N(f) |
| --- | --- | --- | --- | --- | --- | --- | --- | --- |
| Protein free |  | N.A | N.A | N.A | 0.07±0.01 | 177 |  | 24 |
| WT nhTMEM16 | 0.5* | >0.2 | >0.2 | 0.31±0.09 | 0.09±0.03 | 165 | 84.0±6.8 | 24 |
|  | 0 | (8.0±3.0)10^-4^ | (4.7±1.7)10^-4^ | 0.31±0.09 | 0.06±0.01 | 82 | 29.1±5.1 | 24 |
| L302W | 0.5 | (1.8±0.5) ^.^10^-3^ | (0.8±0.2) ^.^10^-3^ | 0.36±0.06 | 0.05±0.01 | 9 | 50.3±15.4 | 6 |
|  | 0# | (3.4±1.2)10^-5^ | (3.4±1.2)10^-5^ | n.d. | n.d. | 6 | 12.7±7.6 | 6 |
| I306W | 0.5 | (2.0±0.8)10^-2^ | (3.5±3.5) ^.^10^-3^ | 0.43±0.02 | 0.06±0.01 | 5 | 68.6±0.7 | 3 |
|  | 0 | (3.5±1.1)10^-4^ | (2.0±0.7)10^-4^ | 0.35±0.01 | 0.07±0.01 | 5 | 20.0±12.9 | 3 |
| N310W | 0.5 | (1.8±0.7)10^-2^ | (4.8±1.9)10^-3^ | 0.41±0.03 | 0.05±0.01 | 6 | 60.7±2.7 | 3 |
|  | 0 | (7.0±0.8)10^-4^ | (3.4±0.6)10^-4^ | 0.36±0.08 | 0.06±0.01 | 6 | 25.0±2.1 | 3 |
| E313W | 0.5 | (5.0±0.3)10^-4^ | (2.6±0.8)10^-4^ | 0.30±0.03 | 0.06±0.01 | 6 | 44.7±8.2 | 9 |
|  | 0# | (1.0±0.3)10^-5^ | (1.0±0.3)10^-5^ | n.d. | n.d. | 6 | 11.1±5.0 | 8 |
| N317W | 0.5* | >0.2 | >0.2 | 0.35±0.03 | 0.08±0.01 | 6 | 75.5±1.3 | 3 |
|  | 0 | (5.0±0.2)10^-4^ | (2.9±0.5)10^-4^ | 0.27±0.01 | 0.06±0.01 | 6 | 3.1±1.6 | 3 |
| F330W | 0.5* | >0.2 | >0.2 | 0.27±0.08 | 0.09±0.01 | 6 | 82.4±1.0 | 3 |
|  | 0 | (7.8±1.1)10^-4^ | (4.0±1.9)10^-4^ | 0.27±0.06 | 0.05±0.01 | 5 | 36.7±4.5 | 3 |
| T333W | 0.5 | (1.7±0.3)10^-1^ | (1.3±0.1)10^-1^ | 0.42±0.01 | 0.06±0.01 | 6 | 77.1±1.5 | 3 |
|  | 0 | (7.3±4.8)10^-4^ | (4.0±3.0)10^-4^ | 0.35±0.02 | 0.05±0.01 | 6 | 26.2±11.7 | 3 |
| L336W | 0.5 | (1.9±0.1)10^-2^ | (0.6±0.1)10^-2^ | 0.30±0.08 | 0.05+0.00 | 6 | 59.5±8.0 | 6 |
|  | 0# | (2.5±1.5)10^-5^ | (2.5±1.5)10^-5^ | n.d. | n.d. | 6 | 13.0±8.7 | 6 |
| V337W | 0.5* | >0.2 | >0.2 | 0.31±0.03 | 0.09±0.01 | 6 | 79.5±5.3 | 6 |
|  | 0 | (2.2±0.2)10^-3^ | (6.2±1.3)10^-4^ | 0.33±0.05 | 0.05±0.00 | 6 | 62.1±6.4 | 6 |
| T340W | 0.5 | (1.4±0.2)10^-3^ | (5.6±0.2)10^-4^ | 0.28±0.01 | 0.05±0.00 | 6 | 55.5±0.5 | 3 |
|  | 0# | (3.4±0.8)10^-5^ | (3.4±0.8)10^-5^ | n.d. | n.d. | 6 | 19.8±11.5 | 3 |
| T381W | 0.5 | (7.9±1.6)10^-4^ | (2.9±0.3)10^-4^ | 0.32±0.06 | 0.06±0.01 | 6 | 57.8±5.7 | 3 |
|  | 0# | (5.4±1.6)10^-5^ | (5.4±1.6)10^-5^ | n.d. | n.d. | 5 | 21.9±6.2 | 3 |
| S382W | 0.5 | (1.2±0.4)10^-3^ | (4.8±1.4)10^-4^ | 0.41±0.09 | 0.06±0.01 | 6 | 70.2±1.2 | 3 |
|  | 0 | (4.5±1.2)10^-4^ | (2.9±0.8)10^-4^ | 0.41±0.09 | 0.05±0.01 | 5 | 40.7±3.6 | 3 |
| A385W | 0.5 | (8.6±1.9)10^-3^ | (4.7±1.4)10-^4^ | 0.39±0.11 | 0.06±0.01 | 6 | 42.3±4.0 | 3 |
|  | 0# | (2.3±1.3)10^-5^ | (2.3±1.3)10^-5^ | n.d. | n.d. | 5 | 11.6±7.4 | 3 |
| R432W | 0.5 | (7.6±2.3)10^-4^ | (4.2±0.9)10^-4^ | 0.37±0.08 | 0.06±0.01 | 11 | 60.7±17.4 | 5 |
|  | 0# | (1.3±0.8)10^-5^ | (1.3±0.8)10^-5^ | n.d. | n.d. | 10 | 10.7±8.2 | 5 |
| N435W | 0.5 | (2.1±0.3)10^-2^ | (0.5±0.2)10^-2^ | 0.36±0.03 | 0.06±0.01 | 6 | 55.6±6.2 | 6 |
|  | 0 | (7.8±1.2)10^-4^ | (5.3±0.7)10^-4^ | 0.36±0.03 | 0.08±0.01 | 6 | 15.2±9.0 | 6 |
| Q436W | 0.5 | (7.0±1.7)10^-4^ | (4.3±0.9)10^-4^ | 0.31±0.14 | 0.06±0.01 | 6 | 24.4±0.8 | 3 |
|  | 0# | (1.5±0.6)10^-5^ | (1.5±0.6)10^-5^ | n.d. | n.d. | 5 | 20.3±2.3 | 3 |
| Y439W | 0.5 | (1.5±0.2)10^-3^ | (4.9±0.5)10^-4^ | 0.33±0.13 | 0.06±0.02 | 5 | 70.6±0.7 | 3 |
|  | 0# | (3.5±1.1)10^-5^ | (3.5±1.1)10^-5^ | n.d. | n.d. | 6 | 35.0±5.8 | 3 |
| F440W | 0.5 | (3.5±1.2)10^-3^ | (1.7±0.3)10^-3^ | 0.37±0.03 | 0.06±0.02 | 6 | 66.5±0.6 | 3 |
|  | 0# | (2.1±2.5)10^-5^ | (2.1±2.5)10^-5^ | n.d. | n.d. | 5 | 12.8±6.8 | 3 |
| E313A | 0.5 | (10.0±2.8)10^-4^ | (3.1±0.9)10^-4^ | 0.31±0.01 | 0.05±0.01 | 6 | 51.3±3.7 | 3 |
|  | 0# | (1.5±0.1)10^-5^ | (1.5±0.1)10^-5^ | n.d. | n.d. | 6 | 10.5±9.6 | 3 |
| E313Q | 0.5 | (9.4±4.1)10^-4^ | (2.2±0.7)10^-4^ | 0.36±0.09 | 0.05±0.01 | 9 | 47.0±4.9 | 9 |
|  | 0 | (3.5±1.8)10^-4^ | (2.3±0.8)10^-4^ | 0.36±0.09 | 0.06±0.01 | 9 | 16.6±10.3 | 8 |
| E313R | 0.5 | (3.0±0.4)10^-4^ | (1.9±0.3)10^-4^ | 0.27±0.01 | 0.05±0.01 | 6 | 26.8±11.5 | 5 |
|  | 0# | (1.6±1.1)10^-5^ | (1.6±1.1)10^-5^ | n.d. | n.d. | 6 | 31.7±6.7 | 5 |
| E313D | 0.5 | 0.16±0.05 | 0.13±0.07 | 0.35±0.03 | 0.07±0.00 | 6 | 62.3±5.6 | 6 |
|  | 0 | (2.1±1.9)10^-3^ | (9.3±7.2)10^-4^ | 0.34±0.03 | 0.04±0.00 | 6 | 14.3±6.2 | 6 |
| R432A | 0.5 | (8.5±3.7)10^-4^ | (5.1±1.3)10^-4^ | 0.25±0.05 | 0.05±0.02 | 9 | 61.7±13.5 | 9 |
|  | 0# | (1.9±1.3)10^-5^ | (1.9±1.3)10^-5^ | n.d. | n.d. | 9 | 20.0±7.6 | 9 |
| R432Q | 0.5 | (6.8±1.3)10^-4^ | (4.7±0.9)10^-4^ | 0.22±0.03 | 0.04±0.00 | 6 | 61.0±15.0 | 6 |
|  | 0# | (1.4±0.9)10^-5^ | (1.4±0.9)10^-5^ | n.d. | n.d. | 6 | 19.2±10.7 | 6 |
| R432L | 0.5 | (6.5±2.3)10^-4^ | (5.0±1.1)10^-4^ | 0.31±0.05 | 0.06±0.02 | 6 | 58.7±1.9 | 5 |
|  | 0 | (0.8±0.4)10^-5^ | (0.8±0.4)10^-5^ | n.d. | n.d. | 6 | 16.3±7.2 | 5 |
| R432E | 0.5 | (4.1±0.3)10^-4^ | (3.3±0.2)10^-4^ | 0.23±0.04 | 0.06±0.01 | 6 | 63.7±11.3 | 6 |
|  | 0# | (1.5±1.6)10^-5^ | (1.5±1.6)10^-5^ | n.d. | n.d. | 6 | 19.0±5.1 | 6 |
| R432K | 0.5 | 0.05±0.01 | 0.04±0.03 | 0.32±0.08 | 0.05±0.01 | 6 | 56.5±6.6 | 6 |
|  | 0 | (5.2±2.0)10^-4^ | (4.1±2.1)10^-4^ | 0.31±0.11 | 0.06±0.01 | 9 | 13.4±11.7 | 6 |
| E318A | 0.5 | (5.5±3.2)10^-4^ | (2.5±0.8)10^-4^ | 0.34±0.05 | 0.04±0.01 | 6 | 38.4±4.5 | 6 |
|  | 0# | (0.9±0.6)10^-5^ | (0.9±0.6)10^-5^ | n.d. | n.d. | 6 | 12.6±11.1 | 5 |
| E318R | 0.5 | (4.8±0.7)10-3 | (6.9±4.5)10^-4^ | 0.33±0.08 | 0.03±0.01 | 6 | 49.6±2.8 | 6 |
|  | 0# | (5.0±1.0)10^-5^ | (5.0±1.0)10^-5^ | n.d. | n.d. | 6 | 7.2±6.8 | 6 |
| E318Q | 0.5 | 0.09±0.02 | 0.09±0.04 | 0.23±0.10 | 0.07±0.01 | 6 | 61.3±9. | 9 |
|  | 0# | (7.5±4.9)10^-5^ | (7.5±4.9)10^-5^ | n.d. | n.d. | 6 | 15.4±8.3 | 9 |
| E313R/  R432E | 0.5 | (4.7±1.1)10-4 | (2.8±0.7)10^-4^ | 0.29±0.02 | 0.06±0.00 | 6 | 23.6±3.5 | 3 |
|  | 0# | (1.4±1.3)10^-5^ | (1.4±1.3)10^-5^ | n.d. | n.d. | 6 | 14.8±1.9 | 3 |
| E313A/  E318A | 0.5 | (3.6±0.1)10^-4^ | (2.4±0.1)10^-4^ | 0.29±0.00 | 0.06±0.00 | 3 | 37.6±3.1 | 3 |
|  | 0# | (2.3±1.3)10^-5^ | (2.3±1.3)10^-5^ | n.d. | n.d. | 3 | 16.0±8.8 | 3 |
| E313A/E318A/R432A | 0.5# | (3.8±1.8)10^-5^ | (3.8±1.8)10^-5^ | n.d. | n.d. | 6 | 20.9±9.4 | 6 |
|  | 0# | (2.3±2.0)10^-5^ | (2.3±2.0)10^-5^ | n.d. | n.d. | 6 | 6.8±2.1 | 6 |
| E313R/E318R/R432E | 0.5# | (2.4±1.5)10^-5^ | (2.4±1.5)10^-5^ | n.d. | n.d. | 5 | 15.6±13.6 | 5 |
|  | 0# | (1.4±0.3)10^-5^ | (1.4±0.3)10^-5^ | n.d. | n.d. | 3 | 10.9±4.5 | 5 |
| Q436A | 0.5 | (1.1±0.8)10^-3^ | (3.7±2.0)10^-4^ | 0.34±0.09 | 0.05±0.01 | 6 | 51.6±4.1 | 6 |
|  | 0# | (2.4±1.4)10^-5^ | (2.4±1.4)10^-5^ | n.d. | n.d. | 6 | 25.5±10.2 | 6 |
| Y439A | 0.5 | (3.9±1.2)10^-4^ | (2.2±0.3)10^-4^ | 0.30±0.09 | 0.04±0.00 | 6 | 61.5±13.1 | 6 |
|  | 0# | (2.0±0.8)10^-5^ | (2.0±0.8)10^-5^ | n.d. | n.d. | 6 | 18.5±6.6 | 6 |

**Supplementary Table 1. Average values of the scrambling rate constants and flux activity for WT and mutant nhTMEM16.** The time course of fluorescence decay was fit to Eq. 4 unless noted by # where Eq. 5 was used instead. The following parameters were derived by fitting the data to Eq. 4: f_0_ is the fraction of empty liposomes, α and β are the forward and backwards scrambling rate constants, γ is the reduction rate constant by dithionite. During these fits L_i_^PF^ was fixed to the value determined in the corresponding batch of protein-free liposomes, across the 177 experiments its mean value was L_i_^PF^=0.53±0.04. * denotes cases where α=β=0.2 was imposed as a constraint as scrambling was too fast to be determined. Where Eq. 5 was used (denoted by #) f_0_ and γ could not be determined (n.d.) as only the linear portion of the decay was fit. The percentage of liposomes containing at least one active channel A, was determined using Eq. 6. “0” Ca^2+^ denotes a concentration <8 nM as previously described ^13^. All data is reported as the mean ± Standard deviation of the parameters derived from the fits, N(s) and N(f) are respectively the number of independent experiments for scrambling and fluxes.

| Protein construct | Number of replicates | Number of lipid flipping events | Direction of lipid flip | total simulation times, µs |
| --- | --- | --- | --- | --- |
| *^a^*WT | 2 | 0 |  | 7 |
| *^b^*WT*^ensemble^* | 24 | 7 | IC to EC | 5 |
| *^a^*A385W | 1 | 1 | EC to IC | 2 |
| *^a^*Q436W | 1 | 0 |  | 2 |
| **TOTAL TIME** |  |  |  | **16** |

**Supplementary Table 2.** Listing of the atomistic MD simulations of nhTMEM16. Simulations carried out on Anton2 are denoted by “*a*” subscript; ensemble simulations carried out with ACEMD are denoted by “*b*” subscript (see Methods for more details). Shown are the protein constructs, the number of independent replicates per construct, the number of lipid flipping events observed, the direction of lipid translocation, and simulation times per construct.

| Dynamic variables used for tICA analysis | |
| --- | --- |
| Interactions between the gates residues | Dynamics of the scrambled lipid |
| T333-Y439 | Z-distance between Lipid and R432 |
| E313-R432 | Minimum distance between Lipid and R432 |
| E318-R432 | Minimum distance between Lipid and E313 |
| Y439-R432 | Minimum distance between Lipid and E318 |

**Supplementary Table 3**. Listing of the dynamic variables used for dimensionality reduction analysis with the tICA approach (see Methods and Results sections for more details).
